# Comparative Evaluation of Anti-Inflammatory Activity of Curcuminoids, Turmerones, and Aqueous Extract of *Curcuma longa*

**DOI:** 10.1155/2013/805756

**Published:** 2013-12-23

**Authors:** Ashish Subhash Bagad, Joshua Allan Joseph, Natarajan Bhaskaran, Amit Agarwal

**Affiliations:** R&D Centre, Natural Remedies, Plot No. 5B, Veerasandra Indl. Area, 19th K.M. Stone, Hosur Road, Electronic City, Bangalore, Karnataka 560 100, India

## Abstract

*Curcuma longa* is widely known for its anti-inflammatory activity in traditional system of medicine for centuries and has been scientifically validated extensively. The present study was conducted to evaluate the anti-inflammatory activity of curcuminoids and oil-free aqueous extract (COFAE) of *C. longa* and compare it with that of curcuminoids and turmerones (volatile oil), the bioactive components of *C. longa* that are proven for the anti-inflammatory potential. The activity against inflammation was evaluated in xylene-induced ear edema, cotton pellet granuloma models in albino Swiss mice and albino Wistar rats, respectively. The results showed that COFAE of *C. longa* at three dose levels significantly (*P* ≤ 0.05) inhibited inflammation in both models, as evidenced by reduction in ear weight and decrease in wet as well as dry weights of cotton pellets, when compared to the vehicle control. The COFAE of *C. longa* showed considerable anti-inflammatory effects against acute and chronic inflammation and the effects were comparable to those of curcuminoids and turmerones.

## 1. Introduction

Inflammation is a transitory biological response of the tissues to harmful stimuli such as injury, exogenous and endogenous antigens, intended to clear or eliminate the stimulus and repair the injured tissue ultimately resulting in regeneration and returning to homeostasis [1]. Though inflammation is a positive defense mechanism of the body, dysregulated and prolonged inflammatory reaction has been well recognized as underlying cause for several disorders, namely, diabetes, allergies, atherosclerosis, obesity, cancer, and pain. Moreover, inflammation dysfunction leading to chronic diseases is contributing to increased health care cost to the society [2, 3].

Nonsteroidal anti-inflammatory drugs (NSAIDs), steroids, and immunosuppressant drugs that have been used conventionally against all forms of inflammatory conditions [4] are associated with adverse effects like ulceration, perforation, gastric irritation, haematochezia [5], angioedema, hepatic failure, headache, hemolytic anemia, hyperglycemia, osteoporosis, immunodeficiency-related problems, and others [6]. Considering these potential adverse effects of these drugs and their limited ability to provide long-term remission, complementary and alternative medicinal products that are generally considered safe are continuously being explored for their anti-inflammatory potential [7].

*Curcuma longa* Linn. (Turmeric) belonging to *Zingiberaceae* family has been widely used as medicine, condiment, and cosmetic worldwide [8, 9] and valued as a functional food because of its health promoting potentials [10]. The rhizome of *C. longa,* a traditional medicine used for centuries in the Indian subcontinent, has been scientifically validated for its antioxidant [11], antimicrobial [12], antiarthritic [13], anticancer [14], carminative, stomachic, tonic, analgesic, hemostatic [15, 16] and anti-inflammatory activities [17]. Most of the studies performed on *C. longa* focused on curcuminoids components which comprised curcumin, demethoxycurcumin, and bisdemethoxycurcumin [18] and the anti-inflammatory effect of *C. longa* was attributed majorly to curcumin [17] acting through the suppression of NF-kappaB and COX-2 activation. The other bioactive components of turmeric, namely, volatile oils, especially turmerones, are also reported to have immunomodulatory and anti-inflammatory activities in few of the studies [19, 20]. Albeit few reports on antiproliferative [21, 22], antidiabetic [23] activities of curcumin-free extract of turmeric are available, anti-inflammatory potential of the same is hardly ever reported.

Curcuminoids and turmerones extracted from *C. longa* are usually used for medicinal as well as cosmetic purposes and indeed the curcuminoids and turmerone-free component of turmeric are often not used in the preparation of turmeric formulations as hardly any information is available on their bioactivity. Moreover, curcuminoids and oil-free aqueous extract of *C. longa*, a spent extract, have not been yet explored for the anti-inflammatory potential. Therefore, the present study was designed to evaluate the anti-inflammatory activity of COFAE of *C. longa* in suitable *in vivo* models, keeping in view that it may help in advancing the scientific understanding and ascertaining a better utility of the extract. The study also focused on comparing the effect of COFAE of *C. longa* with that of curcuminoids and turmerones, the bioactive components of *C. longa *that are already proven for the anti-inflammatory potential.

## 2. Materials and Methods

### 2.1. Animals

Experiments were performed on male albino Swiss mice and albino Wistar rats of either sex bred and reared at Central Animal Facility, Research and Development Centre, Natural Remedies, Bangalore, India. The animals were housed under standard laboratory conditions of 12 h/12 h light/dark cycle at 20–24°C and 30–70% humidity and were provided free access to pelleted rodent feed (M/s Gold Mohur Foods and Feeds, Bangalore, India) and UV purified water. The animal studies were approved by the Institutional Animal Ethics Committee.

### 2.2. Drugs and Chemicals

Dexamethasone and diclofenac sodium were obtained from Cadila Healthcare (India) and Novartis Pharma (India) respectively. Xylene was obtained from Leonid Chemicals (India).

### 2.3. Plant Materials

#### 2.3.1. Curcuminoids

Rhizomes of *C. longa* L. were procured from local market at Bangalore, India, and were authenticated at the National Institute of Science Communication and Information Resources (NISCAIR). A voucher specimen (no. 653) was deposited in the Pharmacognosy Department of R&D Centre, Natural Remedies, Bangalore, India. Coarse ground rhizomes (2 kg) were refluxed with ethyl acetate (8 L) for 3 h on a water bath and filtered. The extraction procedure was repeated two more times. The filtered extract solutions obtained in each step were combined and concentrated by distillation under vacuum at less than 50°C to get a thick paste. The latter was further stirred with petroleum ether (1 : 3, 3 times) at room temperature and the insoluble matter was crystallized using isopropyl alcohol to yield yellow powder of curcuminoids (50 g).

#### 2.3.2. COFAE of *C. longa*

The marc (powdered rhizomes obtained after ethyl acetate extraction) was then extracted with water (8 L, 3 times) at 100°C for 3 hours and filtered. The liquid extracts from three extraction washes were combined and concentrated by distillation under vacuum at 70°C to yield a solution having 20% w/w solids. The concentrated solution was then spray-dried to obtain a free flowing powder (180 g).

#### 2.3.3. Oil of *C. longa*

Coarse ground rhizomes of *C. longa* (5 kg) were suspended in water (100 L) and subjected to steam distillation. The oil layer was separated and passed through anhydrous sodium sulphate to get clear oil (26 mL).

### 2.4. Treatments Schedule

Seventy-two male albino Swiss mice (xylene-induced ear edema model) and seventy-two albino Wistar rats of either sex (cotton pellet granuloma model) were randomly allotted to twelve groups, each consisting of six animals. In xylene-induced ear edema model, the control group received vehicle (water, 10 mL/kg); two other groups of mice were treated with 0.5 and 50 mg/kg body weight of dexamethasone and diclofenac, respectively, as reference standard drugs. The remaining nine groups received three dose levels of curcuminoids, turmerones, and COFAE, respectively. Curcuminoids were administered at 20, 60, and 180 mg/kg mice body weight, whereas turmerones were administered at 0.05, 0.1, and 0.25 mL/kg, while COFAE at 90, 180, and 360 mg/kg mice body weight were administered.

In cotton pellet granuloma model, the control group received vehicle (water, 10 mL/kg); two other groups of rats were treated with 0.5 and 5 mg/kg body weight of dexamethasone and diclofenac, respectively, as reference standard drugs. Curcuminoids were administered at 5, 25, and 125 mg/kg rat body weight, whereas turmerones were administered at 0.05, 0.1 and 0.15 mL/kg, while COFAE at 45, 90, and 180 mg/kg rat body weight were administered to nine groups of rats. Vehicle/reference drugs/test substances were administered by gavage using feeding needle.

### 2.5. Xylene-Induced Ear Edema

The xylene-induced ear edema test was performed as previously described [24]. Male albino Swiss mice weighing 20–30 g (6–8 weeks) were administered with vehicle/drugs/test substances orally 1 h prior to the xylene (50 *μ*L) application to the anterior and posterior surfaces of the right ear topically, while left ear served as control. After 4 h of xylene application, animal was sacrificed; both ears were removed and ear discs of 6 mm diameter were punched out and weighed. The average weight difference between the right and left ear was taken as the measure for inflammatory response.

### 2.6. Cotton Pellet-Induced Granuloma

Albino Wistar rats weighing 150–180 g (6–8 weeks) were completely deprived of food for 1 h before the vehicle/drugs/test substances administration but had free access to water. Rats were anaesthetized and sterile cotton pellets (10 mg) were implanted subcutaneously in axilla and groin regions of rats. The entire procedure was carried out aseptically. Rats were administered vehicle/drug/test substances once daily orally for seven consecutive days. On the eighth day, cotton pellets were meticulously dissected out and dried at 60°C for 24 h. The dry and wet weights of cotton pellet were measured [25]. The percentage inhibition was calculated by using the following formula:

(1) $$\begin{array}{c} \text{Percentage}\;\text{inhibition}=\frac{\left(\text{Control}-\text{Treated}\right)}{\text{Control}}\times 100. \end{array}$$

### 2.7. Statistical Analysis

All the values were expressed as mean ± SEM. The data were analyzed using one-way ANOVA followed by post hoc Dunnett's test. Statistical significance was set at *P* ≤ 0.05.

## 3. Results

### 3.1. Xylene-Induced Ear Edema

**Figure 1:**
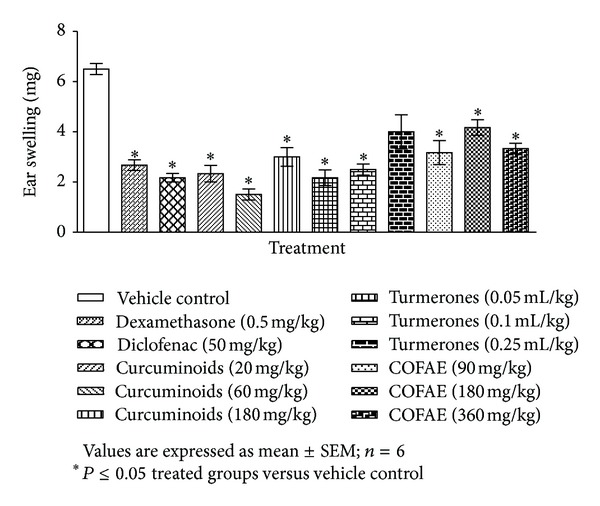
Effect of curcuminoids, turmerones, and COFAE on xylene-induced ear edema in mice.

The average weight of the ears and the percentage inhibition of inflammatory response are presented in Figure 1 and Table 1, respectively. Topical application of xylene caused an evident increase in weight of the right ear when compared to the control left ear in the vehicle control group, whereas the reference drugs, dexamethasone (0.5 mg/kg) and diclofenac (50 mg/kg), that served as positive controls showed significant reduction in the average ear weight in comparison to the vehicle control. Likewise, COFAE of *C. longa* at all the tested doses (90, 180, and 360 mg/kg b.w.), turmerones at the doses of 0.05 and 0.1 mL/kg b.w. and curcuminoids at dose levels of 20, 60, and 180 mg/kg b.w., revealed significant reduction in the average ear weight as compared to vehicle control group.

### 3.2. Cotton Pellet-Induced Granuloma

The average wet and dry weights of cotton pellets along with percentage inhibition are summarized in Table 2. The vehicle control group showed marked inflammatory response manifested as increase in the wet and dry weights of the pellets. The positive controls, namely, dexamethasone and diclofenac, showed significant reduction in both wet and dry weights of the cotton pellets as compared to the vehicle control. Meanwhile, COFAE of *C. longa* at doses of 45, 90, and 180 mg/kg exhibited significant reduction in wet and dry weights of the cotton pellets, as compared to the vehicle control. The percentage inhibition in case of wet weight of pellets which was considered a measure of inhibition of transudative and exudative phases of inflammation was found to be 44.2, 42.6, and 36.9, respectively. And the percentage inhibition in case of dry weight of pellets which was considered a measure of inhibition of proliferative phase of inflammation was found to be 38.7, 34.1, and 32.1, respectively. The curcuminoids at 5, 25, and 125 mg/kg and turmerones at 0.05, 0.1, and 0.15 mL/kg dose levels also revealed significant reduction in wet as well as dry weights of the cotton pellets, as compared to the vehicle control.

## 4. Discussion

Complementary and alternative medicine (CAM) for the treatment of various diseases is gaining popularity globally, at a faster pace since the past two decades [26, 27] and the studies revealed a worldwide market for herbal supplements for the management of inflammatory dysfunction/diseases, which is presently at around 83% and is expected to reach 95% in the forthcoming years [28, 29]. In Ayurveda (a type of CAM), *Curcuma* has a long history of use as an anti-inflammatory agent [30] and has been scientifically validated extensively. However, curcuminoid and oil-free aqueous extract of *C. longa* have not been yet explored for the anti-inflammatory potential and hence the present study was designed to evaluate the anti-inflammatory activity of COFAE of *C. longa* in suitable *in vivo* models to explore biological activity of the extract for use. Furthermore, the effect of COFAE of *C. longa* was compared with that of curcuminoids and turmerones, the bioactive components of *C. longa *that are already proven for the anti-inflammatory potential.

Inflammation constitutes body's response to injury and is characterized by a series of events that mainly occur in three distinct phases. The first phase is caused by an increase in vascular permeability resulting in exudation of fluids from the blood into the interstitial space; the second phase involves the infiltration of leukocytes from the blood into the tissue and third phase is characterized by granuloma formation and tissue repair [31]. Therefore, it is vital to estimate the activities of the test substance in different phases of inflammation, while evaluating the anti-inflammatory effect. Accordingly, COFAE of *C. longa* was investigated for anti-inflammatory potential using acute exudative (xylene-induced ear edema) and chronic proliferative (cotton pellet granuloma) inflammation models [32–36].

Xylene-induced ear edema model is useful for the evaluation of anti-inflammatory topical steroids and nonsteroidal antiphlogistic agents, especially those inhibiting phospholipase A_2_ [37]. Application of xylene induces acute neurogenous edema, which is partially associated with the substance P. Substance P is widely distributed in the central and peripheral nervous system and its release from sensory neurons in the periphery causes vasodilatation and plasma extravasations leading to swelling of the ear, suggesting the role of xylene in neurogenous inflammation [38]. Moreover, the ear edema associated with xylene involves inflammatory mediators such as histamine, kinin, and fibrinolysin [39]. The COFAE of *C. longa* in the present study exhibited significant activity to counter the acute inflammation in the xylene-induced ear edema and the effect was comparable to that of curcuminoids and turmerones. The significant inhibition of xylene-induced ear swelling in mice treated with COFAE of *C. longa* provides a probability that the active principles in the extract could reduce the release of substance P or other inflammatory mediators such as histamine, kinin and fibrinolysin or antagonize the actions.

Chronic inflammation is the reaction arising when the acute response is insufficient to eliminate the proinflammatory agents. Chronic inflammation includes proliferation of fibroblasts and infiltration of neutrophils with exudation of fluid. It occurs by means of development of proliferative cells which can either spread or form granuloma [40]. Cotton pellet granuloma model has been widely used to evaluate the transudative, exudative, and proliferative components of chronic inflammation. Transudate phase causes increase in the wet weight of the cotton pellet while hosting inflammatory response to the implanted cotton pellet between 3 and 6 days causes granuloma formation. Therefore, increase in dry weight is considered as a measure of proliferative component of inflammation [41–43].

In the present study, the wet and the dry weights of the pellets, which correlate with transudative and proliferative (granuloma tissue) components of inflammation, were significantly inhibited by COFAE of *Curcuma* and the effect was equivalent to that of curcuminoids and turmerones. Nevertheless, turmerones showed marginally less anti-inflammatory activity compared to curcuminoids. This reduction in transudate and granuloma formation by COFAE of *C. longa* administration may be correlated with its ability to reduce the number of fibroblasts and the synthesis of collagen and mucopolysaccharides that are involved in the formation of granuloma tissue [44–46].

*C. longa* comprises a group of three curcuminoids such as curcumin (diferuloylmethane), demethoxycurcumin, and bisdemethoxycurcumin, as well as volatile oils (turmerone, atlantone, and zingiberene), sugars, proteins, and resins [17]. Yegnanarayan et al. [47] demonstrated significant anti-inflammatory effect of *C. longa *extracts obtained by petroleum ether, 50% alcohol, and water in both exudative and proliferative inflammation. Subsequently, the extensive research on *C. longa* and curcuminoids specifically on curcumin in the past two decades thoroughly established their anti-inflammatory potential and are reported to downregulate the activity of cyclooxygenase-2 (COX-2), lipoxygenase, and inducible nitric oxide synthase (iNOS) enzymes; inhibit the production of the inflammatory cytokines tumor necrosis factor-alpha (TNF-*α*), interleukins (IL) 1, 2, 6, 8, and 12, monocyte chemoattractant protein (MCP), and migration inhibitory protein; and downregulate mitogen-activated and Janus kinases [17, 48, 49]. Likewise, Funk et al. and Liju et al. have elucidated the anti-inflammatory effects of turmeric essential oils on acute and chronic inflammatory models and suggested an inhibitory effect on release of inflammatory mediators such as histamine, bradykinin, 5-hydroxytryptamine, and prostaglandins as the mode of action. The present study clearly reinstated the anti-inflammatory potential of curcuminoids and turmerones in both acute and chronic inflammatory models.

Curcuminoids and turmerones are usually used for medicinal as well as cosmetic purposes and indeed the COFAE of turmeric is often not used in the preparation of turmeric formulations. Consequently, the present study on COFAE of *C. longa *revealed significant anti-inflammatory potential in both acute exudative and chronic proliferative inflammation models, signifying the presence of bioactive principles in the extract, capable of producing the anti-inflammatory effect.

The whole *C. longa* extract encompasses curcuminoids, volatile oils, and water soluble polysaccharides [50]. However, very few pharmacological studies are available regarding the polysaccharides of *Curcuma* species. A group of polysaccharides isolated from *C. longa*, named ukonan A, B, C, and D, have shown reticuloendothelial system-potentiating activity and anticomplementary activities [51]. Polysaccharides isolated from related species *C. zedoaria* and *C. xanthorrhiza *have been shown to have macrophage-stimulating activity via specific activation of NF-*κ*B [52, 53]. Moreover, recent reports available on polysaccharides from different natural sources indicated their role as free radical scavengers and antioxidants, which has been suggested as the pharmacological basis for prevention of inflammation and atherosclerosis by polysaccharides [54]. Also clinical trial by Madhu et al., [55] that evaluated the efficacy of *Curcuma longa* containing polysaccharides in osteoarthritis patients, indicates the involvement of polysaccharides against inflammation [55]. Hence, the anti-inflammatory effect obtained after COFAE of *C. longa *treatment could be correlated with the presence of polysaccharides and/or any other unexplored active principles. Consequently, there is a definite need for detailed experimental studies to define the precise mechanism of action of COFAE of *C. longa* and to have an elaborative phytochemical study to elucidate the active molecule(s) responsible for the therapeutic effect.

In conclusion, the present study demonstrated potent anti-inflammatory activity of COFAE of *C. longa, *even comparable to that of the proven anti-inflammatory bioactive components of *Curcuma,* namely, curcuminoids and turmerones, in both acute exudative (xylene induced ear edema) and chronic proliferative (cotton pellet granuloma) inflammation models, thereby indicating the possibility of developing COFAE of *C. longa* as a safe and potent anti-inflammatory substance.

## Figures and Tables

**Table 1:** Percentage inhibition of xylene-induced ear edema by curcuminoids, turmerones, and COFAE.

Treatment	Inhibition (%)
Vehicle control	0
Dexamethasone (0.5 mg/kg)	58.9
Diclofenac (50 mg/kg)	66.6
Curcuminoids (20 mg/kg)	64.1
Curcuminoids (60 mg/kg)	76.9
Curcuminoids (180 mg/kg)	53.8
Turmerones (0.05 mL/kg)	66.6
Turmerones (0.1 mL/kg)	61.5
Turmerones (0.25 mL/kg)	38.4
COFAE (90 mg/kg)	51.2
COFAE (180 mg/kg)	35.8
COFAE (360 mg/kg)	48.7

**Table 2:** Effect of curcuminoids, turmerones, and COFAE on cotton pellet-induced granuloma in rats.

	Weight of cotton pellets (mg) (wet)	Inhibition (%)	Weight of cotton pellets (mg) (dry)	Inhibition (%)
Vehicle control (10 mL/kg)	228.6 ± 7.5	0	51.9 ± 1.1	0
Dexamethasone (0.5 mg/kg)	101.2 ± 5.7*	55.7	22.7 ± 1.9*	56.2
Diclofenac (5 mg/kg)	111.0 ± 10.4*	51.4	28 ± 1.7*	46.0
Curcuminoids (5 mg/kg)	143.2 ± 7.4*	37.3	32 ± 2.0*	38.3
Curcuminoids (25 mg/kg)	127.5 ± 12.4*	44.2	31.4 ± 0.6*	39.4
Curcuminoids (125 mg/kg)	132.9 ± 9.6*	41.8	34.7 ± 1.2*	33.1
Turmerones (0.05 mL/kg)	140.1 ± 13.5*	38.7	34.5 ± 1.9*	33.5
Turmerones (0.1 mL/kg)	129.0 ± 3.7*	43.5	33.0 ± 1.0*	36.2
Turmerones (0.15 mL/kg)	142.7 ± 8.6*	37.5	33.0 ± 0.7*	36.2
COFAE (45 mg/kg)	127.4 ± 4.7*	44.2	31.7 ± 1.7*	38.7
COFAE (90 mg/kg)	131.0 ± 6.6*	42.6	34.1 ± 0.9*	34.1
COFAE (180 mg/kg)	144.2 ± 4.8*	36.9	35.2 ± 1.3*	32.1

Values are expressed as mean ± SEM; *n* = 6; *P ≤ 0.05 treated groups versus vehicle control.
